# Influence of contextual factors on most demanding scenarios in under-19 professional soccer players

**DOI:** 10.5114/biolsport.2024.136087

**Published:** 2024-03-06

**Authors:** Rubén-Cipriano Romero-Rodríguez, Enrique Alonso-Pérez-Chao, Carlos Ribas, Daniel Memmert, Miguel-Ángel Gómez-Ruano

**Affiliations:** 1 Facultad de Ciencias de la Actividad Física y del Deporte, Universidad Politécnica de Madrid, 28040 Madrid, Comunidad de Madrid, España; 2 Department of Physical Activity and Sports Science, University Alfonso X el Sabio, 28691 Villanueva de la Cañada, Community of Madrid, Spain; 3 Faculty of Sport Sciences, European University of Madrid, 28670, Madrid, Spain; 4 German Sport University Cologne, Department of Computer Science in Sports and Team/Racket Sport Sciences, Germany, 50933, Köln, Germany

**Keywords:** Team sports, Peak demands, Most demanding scenarios, Load monitoring, Game demands, Contextual factors

## Abstract

This study aimed to compare the most demanding scenarios (MDS) of under-19 professional soccer players during official matches, controlling for contextual factors such as playing position, the level of opponent teams, playing venue, match status, playing surface, pitch size, and playing status of players. A total of 42 players were monitored across 27 games using Global Positioning System (GPS) technology to collect the external loads, including total distance covered, high-speed running, sprint distance, accelerations, and decelerations. MDS were calculated across 1-minute, 5-minute, and 10-minute time windows for each variable. Significant differences were found based on the contextual factors. (i) Opponent level did not show significant differences; (ii) full-backs covered more distance at 1 minute in home games; (iii) midfielders in winning vs. drawing scenarios exhibited higher decelerations at 1 minute but less distance at 10 minutes; (iv) in drawing vs. losing scenarios, central defenders had greater distance at 5 minutes and accelerations at 5 minutes, while midfielders showed greater distances at 1 minute, 5 minutes, and 10 minutes; (v) offensive midfielders and forwards displayed specific performance differences; (vi) no significant differences were found for the playing surface; (vii) regarding pitch size, full-backs covered more distance at 5 minutes on larger pitches, while midfielders covered more distance at 5 minutes and 10 minutes; and (viii) starters generally outperformed non-starter players across various variables depending on the playing positions. The findings suggest that contextual factors have a significant impact on soccer player performance across different playing positions.

## INTRODUCTION

Several studies have investigated the game demands experienced by soccer players, focusing on the average intensity across entire games [1, 2]. However, expressing match intensities as an average disguises the peak intensity period of match play and represents a simplistic perspective [3, 4]. To approach the intensity experienced by players at specific times during the competition, the analysis of shorter epoch durations (e.g., 1 minute and 5 minutes) during match play has been developed [5–8]. Existing literature in this regard has concurred that as the duration of the analysed period increased, the relative intensity decreased for all variables and positions, although this decrease was not consistently linear [9].

The peak demand (PD), which refers to the highest level of activity that players experience for a specific variable during a defined period of time, has been measured in soccer players using various external load variables (e.g., distances and accelerations) and time periods (e.g., 1 minute and 5 minutes) [3, 10, 11]. To quantify the PDs experienced by under nineteen soccer players (< 19 years of age [U19]) during games is particularly important given that the intensities detected can guide optimal training prescription to best prepare players to cope with the most demanding scenarios (MDS) experienced during games [3] and to assist these young players in transitioning to senior-level competitions.

In this regard, various scenarios or conditions inherent in the game [12, 13] should be analysed because they have been shown to influence physical performance [12, 14]. Consequently, external PD may be influenced by numerous contextual factors, which should be taken into account when analysing physical demands [15]. Therefore, it is important to explore the impact of several factors on the peak external values achieved by soccer players during games. This includes investigating the effects of playing position [10, 16], team formation [17, 18], passage duration [9], momentary outcome [19, 20], fixture congestion [21], the number of minutes played by the athlete [2], and the amount of activity completed in the period immediately preceding the MDS [22, 23].

Based on the current literature, the momentary match status was a significant factor in the peak values of a player’s physical performance since, in general, teams when winning reported that players covered more distance, especially at high speeds, compared to drawing matches. Theoretically, achieving a higher external PD during games could indicate that players may be capable of attaining a superior playing pace at key stages across games. In turn, a superior playing pace may indicate an ability to outplay opponents from a physical perspective and improve the likelihood of making successful plays, contributing to team success [24]. In this regard, peak values appeared higher during favourable results (winning) for the total distance, high-speed distance (> 19.8 km/h), and sprint distance (> 25.2 km/h) in most sample durations analysed (1 and 3 minutes) compared to when drawing or losing [20]. Moreover, other studies reported higher peak values for relative distance and Player Load for non-starter players who were winning; the midfielders were those who had higher values in these variables [25]. Alternatively, higher external PD during games may be reflective of game contexts in which a team that is losing at the time might elevate playing intensity to improve the result [24]. Previous studies exploring the impact of team venue (home vs. away) on external PD have indicated higher values for total distance, high-speed distance (> 19.8 km/h), and sprint distance (> 25.2 km/h) across 1-minute, 3-minute, 5-minute, and 10-minute sampling durations when playing away compared to playing at home [20]. However, the existing research has presented conflicting findings. In turn, other studies suggest that home teams are likely to experience greater peak external intensities compared to away games [12, 26].

Nevertheless, research regarding the influence that these contextual factors may have on the peak values in professional football is scarce given the limited number of studies available to date. Based on the foregoing, the need to consider situational factors in the analysis of MDS and try to interpret how these variables can affect the responses of athletes would seem indispensable [27]. Therefore, this study aimed to quantify and compare the external PD encountered during official games based on various contextual variables including playing position (central defender, full-backs, midfielders, wide midfielders, and offensive midfielders, and forwards), opponent level (high, medium, and low level), team venue (home or away), momentary outcome (winning, drawing and losing), playing surface (artificial or natural grass), pitch size (large, medium, small) and player role (starter and non-starter). Based on existing data [20], it was hypothesized that external PD may be influenced by several contextual factors.

## MATERIALS AND METHODS

### Sample

Male professional soccer players from the same team competing in the highest national division of a U19 Spanish soccer competition (n = 42, mean ± standard deviation [SD]: age 18.0 ± 0.4 years, height 181.4 ± 6.8 cm, body mass: 70.1 ± 5.6 kg) were monitored during 34 official games. Players were considered to be professional as they had an exclusive dedication to this sport with a frequency of 6 weekly sessions (5 practices and 1 game).

For inclusion in the study, players had to complete at least a half-time (45 minutes of playing time). Then, each game only included data from those players who played at least 45 minutes, since substitutes can have higher outputs than starting players, likely because of pacing strategies [9]. Additionally, players who participated in 2 different positions during the same game (e.g., a player who started playing as a midfielder and ended up playing as a wide midfielder) were excluded from that specific game, since changes in positions have an impact on the physical performance of players [28]. In turn, goalkeepers were excluded from the analysis due to the distinct nature of their activity-demands profile. Any game in which any team played with numerical superiority, either due to player send-off, a red card, or injuries, as well as blowout games (a difference of more than five goals in favour of a team), were excluded from the analysis. These exclusion criteria have been justified in previous similar studies as these situations could potentially impact the demands and, particularly, the physical intensity of the match [29, 30]. As a result, four initially recruited players and seven monitored games did not meet the inclusion criteria and were therefore excluded from the final analyses. This led to a retained sample of 38 players and 27 games for the study. Considering entire game analyses, n = 289 game samples across the n = 38 players were included in the final analyses.

The team formation was 4-2-3-1. Players were deployed in each match as follows: two central defenders, two full-backs, two midfielders, two wide midfielders, one offensive midfielder, and one player in the forward position. Depending on the substitutions made by the coach in each match, and the timing of these substitutions, there might be variations in the number of records for each position. For instance, if a forward player was replaced at halftime by another of the same position, even though the game system only fielded one player of that position per game, that specific match could include the analysis of up to two players in the same position, given that both met the criteria by playing at least 45 minutes.

### Procedures

This observational investigation was conducted across a 12-month period throughout the 2020–2021 season. The devices (Vector S7; Catapult Sports, Melbourne, Australia) were calibrated according to the manufacturer’s instructions, which consisted of placing the device on a flat surface, turning the units on without surrounding magnetic devices, and finally, waiting for 60 s. Then, the units were placed in the back of a specific chest vest. Each player wore a device in a bespoke pocket within a vest positioned on the upper thoracic spine between the scapulae. The devices contained a triaxial accelerometer (± 16 g, 100 Hz), magnetometer (± 4.900 μT, 100 Hz), gyroscope (up to 2,000 deg/s, 100 Hz), and a Global Positioning System (GPS) receptor (10 Hz), and all units were updated with the same firmware version (6.5.0). The technology used in this study has been supported as valid in measuring distance [31, 32], speed, accelerations, and decelerations [32, 33], while similar GPS technology is reliable (coefficient of variation (CV) < 5%) in measuring distance and speed variables [31]. All participants were familiarized with the devices as part of their day-to-day training and playing practices. Each device was turned on ~20–40 minutes before the warm-up preceding each game. Players wore the same assigned unit throughout the course of the data collection period to avoid inter-device variation in external load data outputs [34, 35].

To establish the external PD for each match observation, first, the raw data were extracted in 1-s intervals for each player. Data were then exported to a custom-built Microsoft Excel (version 16.0; Microsoft Corporation, Redmond, WA) spreadsheet for further analysis. Data were analysed across different time windows (1 minute, 5 minutes, and 10 minutes) using rolling averages to find the peak value for each variable across each duration. This rolling average method entails computing averages for a designated window or interval of consecutive data points as the window progressively moves through the dataset. This approach is frequently employed when assessing MDS [4] and has been previously used in several studies [6, 10, 19, 36, 37]. The data were analysed separately for the first and second halves of each game, with the highest values for each variable in a specific game used for game analysis (i.e., the highest value for each variable across any moment of the game in a specific game was taken as the value for that game).

### Physical variables

Variables analysed were selected based on previous publications [6, 25]. Thus, external PDs were calculated for several external physical load variables including total distance (m) covered (TD) and distance (m) covered in different intensity zones including high-speed running (HSR) > 21 km/h, as previously used in soccer research [30, 38] and sprint distance (SD) > 24 km/h. Furthermore, accelerations (ACC) (count) performed > 2 m · s^−2^ (dwell time: 0.3 seconds), and decelerations (DEC) (count) performed < -2 m · s^−2^ (dwell time: 0.3 s) were also measured. These dwell times were chosen given that values between 0.3 and 0.4 s have been identified as the most readily used in team sports settings [39].

### Contextual variables

The contextual variables analysed were playing position (e.g., central defender, full-backs, midfielders, wide midfielders, offensive midfielders, and forwards), opponent level (e.g., high, medium, and low level), team venue (e.g., home, and away), match status (e.g., winning, drawing, and losing), playing surface (e.g., artificial, or natural grass), pitch size (large, medium, small) and player role (starter and non-starter).

– **Playing position:** To explore potential differences based on playing positions, the total sample was categorized into six typical soccer roles: central defender (CD) (n = 6, 65 samples), full-backs (FB) (n = 8, 77 samples), midfielders (MF) (n = 9, 69 samples), wide midfielders (WMF) (n = 8, 55 samples), offensive midfielders (OMF) (n = 6, 62 samples) and forwards (FW) (n = 5, 59 samples).
– **Opponent level:** Concerning the opponent level, we examined differences in peak values when the reference team played against high-level teams (ranked in the top 6 league positions), medium-level teams (ranked 7^th^ to 13^th^ in the league), and low-level teams (ranked in the bottom 7 of the league). These categories are similar to those reported previously [13, 14, 40].
– **Team venue:** Comparisons according to playing venue were made between games that were played at home (13 games, 76 samples) and away (14 games, 84 samples).
– **Match status:** Match status was defined as winning, drawing, or losing in relation to the number of goals scored and conceded by the sampled team at the time of data entry [41].
– **Playing surface:** Comparisons according to playing surface were made between games that were played on natural grass (14 games, 82 samples) and games that were played on artificial grass (13 games, 78 samples).
– **Pitch size:** Pitch dimensions were classified as large size (≥ 106 × 68 m; 7208 m^2^); medium size (> 93 × 55 m; 5115 m^2^ and < 106 × 68 m; 7208 m^2^), and small size (< 93 × 55 m; 5115 m^2^).
– **Player role:** was established as a starter (156 samples) or non-starter (83 samples). For inclusion in the study, players had to complete at least a half-time (45 minutes of playing time). Then, only benching players who started from the beginning of the second half were considered non-starter players and accordingly included in the study.

### Statistical analysis

The Kolmogorov-Smirnov normality test was performed to check data normality assumptions. The linear mixed model (LMM) was used to detect significant differences within groups (high level vs. medium level vs. low level; drawing vs. winning vs. losing; large vs. medium vs. small) and to test significant differences within the other conditions (playing position, opponent level, team venue, match status, playing surface, pitch size and player role) depending on players’ position. Cohen’s effect size (ES) and the mean difference with 95% confidence intervals (CI) were determined for all pairwise comparisons and interpreted as trivial = < 0.20; small = 0.20–0.59; moderate = 0.60–1.19; large = 1.20–1.99; and very large = > 2.00 [42]. All analyses were conducted using IBM SPSS for Windows (version 23, IBM Corporation, Armonk, New York), except ES, which was calculated using a customized Microsoft Excel spreadsheet (version 16.0, Microsoft Corporation, Redmond, WA). The significant level was set at p < 0.05.

## RESULTS

**FIG. 1:**
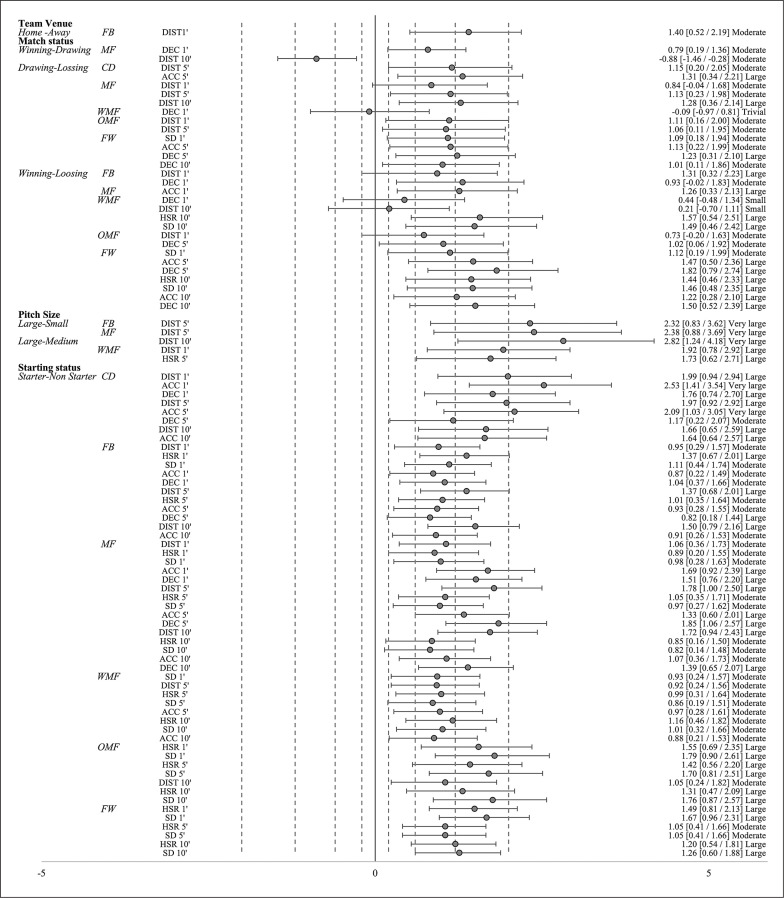
Standardized effect size magnitude [95% confidence interval] between conditions for all variables depending on playing position and time window (1’, 5’ and 10’). Notes: The dotted lines represent the effect size magnitude thresholds from trivial to large (see Methods), playing positions: CD = central defender, FB = full- backs, MF = midfielders, WMF = wide midfielders, OMF= offensive midfielders and FW = forwards. External physical load variables: TD = total distance covered, HSR = high-speed running (> 21 km/h), SD = sprint distance (> 24 km/h), ACC = accelerations (> 2 m · s^−2^) and DEC = decelerations (< -2 m · s^−2^)

Mean comparisons according to the condition for each time window depending on players’ position are presented in Figure 1.

### Opponent level

No significant differences (p > 0.05) were observed in the comparison between varying opponent levels (high vs. medium vs. low).

### Team venue

In the comparison between home and away games, FB notably covered a greater distance (DIST) at 1’ (p = 0.04; ES = 1.40). Conversely, no significant differences (p > 0.05) were observed in the other comparisons between home and away games.

### Match status

In the comparison between winning and drawing statuses, MF showed significantly higher DEC 1’ (p = 0.04; ES = 0.79) and notably lower DIST 10’ (p = 0.02; ES = -0.88).

Regarding the comparison between drawing and losing statuses, CD demonstrated significantly greater DIST 5’ (p = 0.02; ES = 1.15), and ACC 5’ (p = 0.01; ES = 1.31). Meanwhile, MF displayed notably higher DIST at 1’ (p = 0.01; ES = 0.84), DIST 5’ (p < 0.01; ES = 1.13), and DIST 10’ (p < 0.01; ES = 1.28). OMF exhibited higher DIST at 1’ (p = 0.01; ES = 1.11) and DIST 5’ (p = 0.04; ES = 1.06). FW showed significantly greater SD 1’ (p = 0.03; ES = 1.09), ACC 5’ (p < 0.01; ES = 1.13), DEC 5’ (p < 0.01; ES = 1.23), and DEC 10’ (p < 0.01; ES = 1.01) for drawing games compared to losing. However, WMF displayed notably lower DEC 1’ (p = 0.03; ES = -0.09) when comparing drawing to losing status.

In reference to the comparison between winning vs. losing status, FB disclosed considerably higher DIST 1’ (p = 0.02; ES = 0.93) and DEC 1’ (p = 0.02; ES = 1.31); MF revealed substantially higher ACC 1’ (p = 0.01; ES = 1.26); WMF revealed significantly greater DEC 1’ (p < 0.01; ES = 0.44), DIST 10’ (p = 0.01; ES = 0.21), HSR 10’ (p = 0.01; ES = 1.57), and SD 10’ (p = 0.03; ES = 1.49); OMF expressed substantially larger DIST 1’ (p < 0.01; ES = 0.73) and DEC 5’ (p = 0.04; ES = 1.02); and FW exhibited considerably higher SD 1’ (p = 0.02; ES = 1.12), ACC 5’ (p < 0.01; ES = 1.47), DEC 5’ (p < 0.01; ES = 1.82), HSR 10’ (p = 0.01; ES = 1.44), SD 10’ (p = 0.01; ES = 1.46), ACC 10’ (p < 0.01; ES = 1.22) and DEC 10’ (p < 0.01; ES = 1.50) during winning games compared to losing (Figure 1). No significant (p > 0.05) differences were observed in the remaining comparisons.

### Playing surface

Regarding the comparison between different playing surfaces (natural grass vs. artificial grass), no significant differences were observed (p > 0.05).

### Pitch size

When comparing different pitch sizes (large vs. small), FB revealed significantly larger DIST 5’ on larger pitches (p = 0.04; ES = 2.32), while MF showed greater DIST 5’ (p = 0.03; ES = 2.38) and DIST 10’ (p = 0.01; ES = 2.82).

In the comparison between large vs. medium pitch size, WMF revealed appreciably higher DIST 1’ (p = 0.03; ES = 1.83) and HSR 5’ (p = 0.03; ES = 2.04).

No significant (p > 0.05) differences were observed in the remaining comparisons.

### Starting status

Regarding the comparison between starter vs. non-starter role, CD displayed significantly higher values for DIST 1’ (p < 0.01; ES = 1.99), ACC 1’ (p < 0.01; ES = 2.53), DEC 1’ (p < 0.01; ES = 1.76), DIST 5’ (p < 0.01; ES = 1.97), ACC 5’ (p < 0.01; ES = 2.09), DEC 5’ (p = 0.03; ES = 1.17), DIST 10’ (p = 0.00; ES = 1.66), ACC 10’ (p < 0.01; ES = 1.64); FB expressed significantly larger values for DIST 1’ (p = 0.02; ES = 0.95), HSR 1’ (p < 0.01; ES = 1.37), SD 1’ (p = 0.01; ES = 1.11), ACC 1’ (p = 0.03; ES = 0.87), DEC 1’ (p < 0.01; ES = 1.04), DIST 5’ (p < 0.01; ES = 1.37), HSR 5’ (p = 0.01; ES = 1.01), ACC 5’ (p = 0.01; ES = 0.93), DEC 5’ (p < 0.01; ES = 0.82), DIST 10’ (p < 0.01; ES = 1.50), ACC 10’ (p < 0.01; ES = 0.91); MF showed significant differences for all variables across all time windows; WMF showed substantially higher SD 1’ (p = 0.02; ES = 0.93), DIST 5’ (p = 0.04; ES = 0.92), HSR 5’ (p = 0.01; ES = 0.99), SD 5’ (p = 0.04; ES = 0.86), ACC 5’ (p = 0.01; ES = 0.97), HSR 10’ (p < 0.01; ES = 1.16), SD 10’ (p = 0.01; ES = 1.01), ACC 10’ (p = 0.03; ES = 0.88); OMF got significantly greater HSR 1’ (p < 0.01; ES = 1.55), SD 1’ (p < 0.01; ES = 1.79), HSR 5’ (p < 0.01; ES = 1.42), SD 5’ (p < 0.01; ES = 1.70), DIST 10’ (p = 0.03; ES = 1.05), HSR 10’ (p < 0.01; ES = 1.31) and SD 10’ (p < 0.01; ES = 1.76); and FW reached significantly higher HSR 1’ (p < 0.01; ES = 1.49), SD 1’ (p < 0.01; ES = 1.67), HSR 5’ (p < 0.01; ES = 1.05), SD 5’ (p < 0.01; ES = 1.05), HSR 10’ (p < 0.01; ES = 1.20) and SD 10’ (p < 0.01; ES = 1.26) when starting the games compared to when not starting (Figure 1). No significant (p > 0.05) differences were found in the remaining positions across each variable.

## DISCUSSION

This study aimed to quantify and compare the external peak demands (PD) encountered by under-19 professional soccer players during official games according to different contextual variables such as playing position, opponent level, team venue, momentary outcome, playing surface, and player role. Based on the results obtained, it can be confirmed that contextual variables had a relevant influence on PD in official matches, in the different variables studied, and for different playing positions, as had already been suggested in previous research [27]. In addition, our results corroborate the previously demonstrated differences in PD concerning playing position, as explored in previous research [9, 10, 37].

Regarding the team venue (home or away matches), playing as the home team resulted in higher values in the PD of total distance covered and distance covered at different speed ranges (> 21 and > 24 km/h) compared to playing away, especially in the position of FB for the period of 1 minute. This aligns with the findings of other studies that confirmed the higher physical demands in games played at home compared to away games, primarily for the variable TD and in the absolute values of completed matches [12, 43, 44]. However, some studies specifically analysed PD and reported the opposite [20, 45]. For instance, higher values for PD in TD, HSR, and SPD were reported during away games compared to home games in periods of 1’, 5’, and 10’. Nevertheless, the results are in line with previous findings [45] where PD for ACC and DEC were significantly higher in away games, especially for FW in periods of 1’ and 10’, coinciding with the 1’ periods. These differences could be attributed to the interacting effects of contextual factors, such as match status, field dimensions, and spectator influence [46].

Concerning the playing surface, no significant differences were observed (p > 0.05). Although we lack comparable data in the literature specifically related to PD regarding this factor, what appears evident is that the two surfaces have different mechanical properties, and the playing surface can alter the demands of the competition and the physiological response in football-specific activity [47]. Research suggests that physical performance in terms of specific efforts [48] and agility tests [49] was better on artificial turf compared to natural grass, accompanied by lower physiological load, and perceived fatigue [47]. As for the physical performance of full games, it was found that TD and very high-intensity running (VHIR; running speed from 16.1 to 19 km/h) covered by players on artificial turf were significantly higher compared to natural grass [50]. However, it should be noted that this study did not analyse PD, and the sample consisted of U14 players in a different competition, which could potentially explain these variations. Based on these findings, it could be assumed that playing on natural grass could increase fatigue, impacting a player’s running activity and reducing their performance in the game. This may lead to a slower pace of play and fewer high-intensity runs [2], although these can also be influenced by other contextual factors (e.g., the level of opponent teams) [51].

The distinction between being a starter or a non-starter in a match also resulted in differences in the PD for most of the analysed variables and positions. Noticeable differences (large or very large) between starters and non-starters were observed for CD positions in the variables ACC (1’, 5’ and 10’), DECC (1’, and 5’), DIST (1’, 5’ and 10’); FB in DIST (1’, 5’ and 10’), HSR (1’ and 5’), SD (1’), ACC (1’. 5’ and 10’) and DEC (1’ and 5’); MF (all variables across all time windows); OMF in HSR (1’, 5’ and 10’) and SD (1’, 5’ and 10’); FW in HSR (1’, 5’ and 10’), and SD (1’, 5’ and 10’); WMF in DIST (5’), HSR (5’ and 10’) and SD (1’, 5’ and 10’), favouring the starting players. This suggests that starting players, by completing more uptimes, are more exposed and likely to be exposed to PD. These results are partially consistent with previous research [19], where starter players showed higher PD for TD for all playing positions when compared with non-starter players. However, in this research, non-starter players showed higher peak values for HSR PD for the 5’ period compared to starter players [19].

Previous studies found that the status of playing as a starter or non-starter did not have an impact on total distance, but it did have an influence on the high-speed distance (> 19.8 km/h) and sprinting distance (> 25.2 km/h), with starting players having higher values in 1’, 3’, 5’, and 10’ periods [9]. The data from the present study support this notion, although the threshold values differ. On the other hand, in a recent study non-starters tended to produce higher peak values for the TD variable in the 3’ window. However, this same research also reflected that the lowest values of PD in TD happened in the final part of the matches for most players, although these are the minutes in which non-starting players usually participate [36]. Nevertheless, one of the findings was that the SD variable tended to appear in those final moments, possibly due to the presence of players who entered the field without fatigue or the influence of contextual factors and/or tactical considerations that would keep them with an orderly participation [25]. An example of this influence was reflected in the position of MFs, who had higher values of PD for TD and Player Load when the team was winning for non-starting players compared to starters [25].

Based on the analysis of PD regarding the standard of teams, the present study demonstrated that PD for TD was higher when playing against high-level teams, especially for WMF and OMF (5’ and 10’ minutes), SD, and MF (1’ and 5’ minutes). However, FB showed higher peak values for ACC (5’ minutes) when playing against medium-level teams. These results correspond to most research on this factor [43, 52, 53] where higher average values for TD and HSR were also found when playing against medium or high-level teams compared with low-level teams [26, 54]. The opposite has also been reflected. Apart from the maximum speed reached, the high-level team showed greater distance covered in matches against low-level opponents, in the variables total TD, average speed (km/h), low intensity running (LIR = > 11.01, and < 14 km/h), medium intensity running (MIR = > 14.01 and < 19 km/h) and high intensity running (HIR = > 19.01 and < 23 km/h) than against high-level opponents [26].

When comparing opponent levels, no significant differences (p > 0.05) were observed in the comparison between varying opponent levels (high vs. medium vs. low). These results do not align with the findings of previous research [43, 55] where higher values for TD and HIR were observed against high-level teams as opposed to low-level teams. These results diverge from earlier studies [e.g. 53], particularly concerning variables related to ACC and DEC. Varley, Gregson, et al. (2017) also reported higher demands in HSR without the ball for medium-level teams compared to low-level teams, along with higher demands in HSR with the ball for low-level teams compared to medium-level teams [56]. The exploration of the relationship between ball possession and the physical demands of the game in this research is novel, as previous studies have not considered this factor. Additionally, there is a lack of research on how the level of the opposing team influences the physical demands, making this study potentially ground-breaking in addressing this gap.

Based on the match status, our results showed that MF exhibited a significant increase in deceleration (DEC 1’) during winning games compared to drawing games. However, they showed notably lower distance covered at 10 minutes (DIST 10’) during winning games. This suggests that midfielders contribute more to the deceleration aspect in winning scenarios but cover less ground over an extended period. These results deviate from the findings of previous research [45] which which pointed out larger distances covered during PD windows of 1’, 3’, and 5’, being significantly higher during moments when the team was drawing compared to moments when they were winning.

Concerning tied versus losing scenarios, the PDs were also notably higher during tied results for almost all playing positions and variables. This aligns with previous findings, where PDs were observed to be elevated during tied situations for TD (1’, 3’, and 5’) [20, 45]. In the comparison between winning and losing, our analysis revealed higher PDs across almost all variables and playing positions when the team was in a winning position. Particularly significant differences were noted for the FW, WMF, and OMF (HSR 5’ and 10’). This may suggest that, during winning situations, attacking positions adopt a more aggressive pressing attitude and exert increased efforts in the opponent’s field. This aligns with a recent study indicating that attacking players (FW, WMF, and MF) exhibited higher demands in HSR (> 21 km/h), very HSR (VHSR = > 21 and < 24 km/h), and SD (> 24 km/h) when their team was winning [57]. To date, research has commonly shown that there is greater physical performance in terms of total game demands when the result is unfavourable compared to drawing or winning [14]. In terms of PD, teams demonstrated higher game demands in the variables of TD, HSR, and SD when they won, as opposed to drawn games [20]. In this regard, peak values appeared to be higher during favourable results (winning) for total distance covered, HSR (> 19.8 km/h), and SD (> 25.2 km/h) in most of the analysed sample durations (1’ and 3’) compared to drawn or lost games [20].

Regarding the size of the playing pitch, the results reveal notable variations in performance metrics across different pitch sizes. When comparing large versus small pitches, FB demonstrated significantly larger DIST 5’ on larger pitches (ES = 2.32), whereas MF exhibited greater DIST 5’ (ES = 2.38) and DIST 10’ (ES = 2.82) on larger playing areas. The factor of playing pitch size is mainly related to the “individual playing area” or “area per player” (ApP) [58], which has been widely studied and shown to directly affect the conditioning demands of the players [59, 60]. In our analysis, when comparing a large-sized pitch with a medium-sized one, WMF showed significantly higher DIST 1’ (ES = 1.83) and HSR 5’ (ES = 2.04) on larger pitches, indicating a consistent trend towards enhanced performance in these variables. When comparing large-sized pitches vs. small-sized pitches, no significant differences (p > 0.05) were observed. This suggests that the transition from a medium-sized pitch to a small-sized pitch may not have a pronounced effect on the observed performance metrics.

Despite this, the overall trend continues to suggest a correlation between pitch size and performance metrics, with larger pitches generally favouring higher PD across various playing positions and variables. These results align with the findings of previous studies [60, 61], indicating that a larger playing area corresponds to increased demands for HSR. To meet these demands, playing areas with larger ApP are necessary, with the order of importance being SD > HSR > TD. Notably, this heightened demand associated with larger playing areas did not have a significant impact on measures of ACC and DEC [61]. Furthermore, in line with earlier research conducted in various small sided games (SSGs) [62], MFs exhibited higher values in medium-sized dimensions (SSG 7 v 7+3; ApP 61.4 m^2^) and large dimensions (SSG 8 v 8+3; ApP 73.7 m^2^) compared to small dimensions (SSG 4 v 4+3 ApP 21 m^2^ & SSG 5 v 5+3 ApP 38.5 m^2^). These consistent findings across different studies provide additional support to the idea that pitch dimensions, particularly the individual playing area, play a crucial role in influencing player performance and conditioning demands.

To our knowledge, there is no existing research specifically related to PD or official match demands concerning this factor. Considering the competitive regulations established by federations, it might not seem logical to consider this aspect, as these regulations typically mandate standardized measurements for playing surfaces, assumed to be equal or meeting minimum standards. However, this situation differs for the top national U-19 soccer category in Spain. In this case, the dimensions of the fields where official matches are played vary significantly, ranging from large to medium or small dimensions. This leads to a notable alteration of the relative area per player, resulting in a potential fluctuation of over 100 m² per player between different playing fields within the same league. Such variations can have significant implications due to the conditioning differences they introduce. A recent meta-analysis [59] showed that, in spaces larger than 100 m² per player, each increase of 25 m^2^ of ApP increased the exposure to HSR by an average of 2.8 to 1.9 m · min^−1^, in VHSR it increased between 0.6 and 0.9 m · min^−1^ and in SD it increased between 0.1 and 0.3 m · min^−1^. Hence, disparities in the playing area exceeding 100 m² per player will lead to significant distinctions, as our results identified.

The limitations of this study should be considered when interpreting our results. First, only external load variables were monitored, and therefore internal PDs (e.g., heart rate, rating of perceived exertion) were not explored; they may show different patterns to those observed in our study for external demands. Secondly, the sample size employed in this study was relatively small, as it involved recruiting only one soccer team. Consequently, these findings may not be applicable to all soccer teams due to potential variations in success rates, tactical strategies, playing pace, and player fitness likely to vary within and between competitions.

### Practical applications

Based on the obtained results, several practical applications can be suggested depending on the orientation, games per week, individual necessities, objectives, and different contextual variables. Coaches and strength conditioning professionals should devise strategies to expose players to the MDS during practice sessions, aiming to prepare athletes to handle the most intense demands of the game. Moreover, by analysing the physical demands of different playing positions, coaches can discern the strengths and weaknesses of individual players. This information allows them to make informed decisions when selecting players for specific games. Additionally, understanding the specific physical demands associated with different playing positions and opponent levels empowers coaches to design training programmes tailored to the specific needs of the team.

Concerning return-to-performance processes, considering the higher PD when a player is returning to play allows coaches and medical staff to implement necessary precautions. Gradually reintroducing the player to the physical demands of the game may involve strategies such as reducing playing time, increasing rest periods, and implementing a structured tapering programme. This approach aims to enhance the physical conditioning and reduce the risk of re-injury. In this context, natural grass surfaces seem to demand additional muscular and metabolic capacity from players in football practice. Therefore, it is advisable for coaches to tailor specific player training regimens to the characteristics of the field surface. This adaptation could be a crucial consideration in the decision-making process.

## CONCLUSIONS

Numerous contextual factors, including the playing surface, starting status, opponent level, and match status, can exert a significant influence on the performance of soccer players across various playing positions.
